# Diet of Parturient Women

**Published:** 1871-03

**Authors:** 


### DIET OF PARTURIENT WOMEN.

Dr. Hugh Miller calls attention to the very vague instructions given by obstetric writers on this subject. Particulars of a case were given, in which careful nourishing diet given during utero-gestation enabled the patient in her last confinement to escape suffering from uterine inertia. From an examination into the physiology of the changes in the uterus and breast, Dr. Miller believed that the fat-cells existing in abundance in the milk during the first few weeks were due to the changes in the womb after parturition; that the disintegrating uterus was broken up into fat-cells, which were absorbed by the blood, and through the circulation were secreted by the mammary glands. Hence a heat-forming diet was neither necessary nor was indicated, and at times might be positively injurious; whereas a flesh-forming diet by maintaining the strength, enabled the woman to make up for the waste of tissue during labour, gave her support, and maintained the vigor of her body while the further changes were going on. The author had found great benefit through selecting the parturient woman's diet from as nearly as possible the kind of food which she was in the daily habit of taking, giving it in a liquid form and in diminished quantity. The advantages in adopting a nourishing diet to the mother he believed to be: 1. Maintaining her muscular strength. 2. Avoiding irritation to the mammary glands and enabling her to suckle sooner. 3. Securing a quicker and better recovery.

Dr. Robert Barnes says that he has noticed great mischief brought about by giving nutritious diet too soon after parturition. He did not say that such diet was not necessary; but there was a prevalent tendency to go too far, and to load the stomach before the patient was able to bear it. The system after parturition required repose, and that in consequence of the changes that took place little food was at first required. It was not desirable to give stimulants at all, and certainly not solid food.—*British Med. Journal*, Oct. 1, 1870.

